# Total team protection during endovascular aortic repair utilizing a novel radiation shielding system

**DOI:** 10.1016/j.jvscit.2026.102172

**Published:** 2026-02-05

**Authors:** Joshua Meredith, Kody Kasten, Patrick Muck

**Affiliations:** Department of Vascular Surgery, Good Samaritan Hospital, Cincinnati, OH

**Keywords:** Radiation safety, Rampart, Total team protection

## Abstract

Vascular surgery has evolved to include a large proportion of minimally invasive endovascular procedures. These procedures are better tolerated by patients, but can be taxing for the operator with less than adequate ergonomics and high effective radiation doses. Lead aprons do not protect the entire body and cause significant strain on the spine. Traditional lead shields are difficult to adequately position to optimize visualization and operator protection. Novel radiation protection systems provide stable protection for the entire team. Here we present a case using a novel protection system allowing the entire team to operate without traditional lead apron shielding.

With the advent of endovascular therapy, perioperative complication and mortality rates improved in many cases. However, one new danger is radiation exposure both to the patient and to the operator. As low as reasonably achievable (ALARA) was begun in 1974 in an effort to initiate protection regulations primarily in nuclear engineering.^1^ However, the same principles apply to endovascular interventions. Radiation scatter is a major concern for operator exposure. Appropriate collimation, decreasing fluoroscopy time, digital subtraction angiography, and avoiding steep oblique angulation are also methods of decreasing exposure. However, with the increasing complexity in endovascular procedures, time of exposure and difficulty using traditional shields result in high effective doses to the operator. Wearable lead does not mitigate exposure to the uncovered portions of the body, including the eyes, head, and arms in particular. Furthermore, lead apron protection weighs as much as 25 lbs, leading to increased axial loading and operator discomfort and orthopedic injuries over time.^2^^,^^3^ A single-center prospective study revealed that, despite optimizing standard protective measures for trainees, monthly doses were still 272 mRem, compared with 936 before multiple system optimizations.^4^ This finding highlights the need for alternate methods of protection.

The Rampart Guardian system (Rampart IC) was developed to allow teams to avoid the cumbersome shields and wearable lead. Boasting a 99% reduction in scatter radiation and 20 times greater protection than standard aprons and shields, they validated their device in two randomized controlled trials. These trials showed a significant decrease in radiation exposure of ≤100% for the operator and team allowing lead-free procedures.^5^^,^^6^ The below case is, to our knowledge, the first endovascular aortic repair performed with the Guardian and the entire operative team free of traditional wearable shielding during the procedure.

## Case report

**Fig 1:**
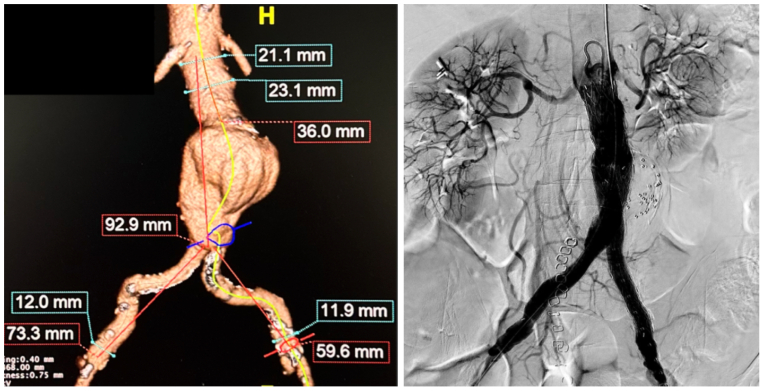
Preoperative three-dimensional rendering of an infrarenal abdominal aortic aneurysm with measurements and postoperative completion angiogram.

**Fig 2:**
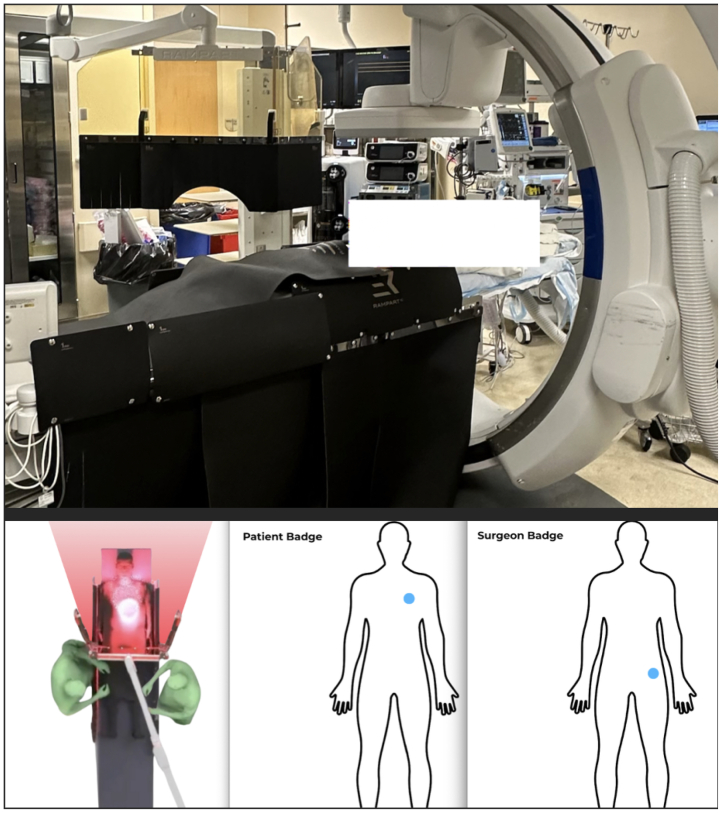
Rampart Guardian, Bunker, and Shadow setup before endovascular aneurysm repair.

**Fig 3:**
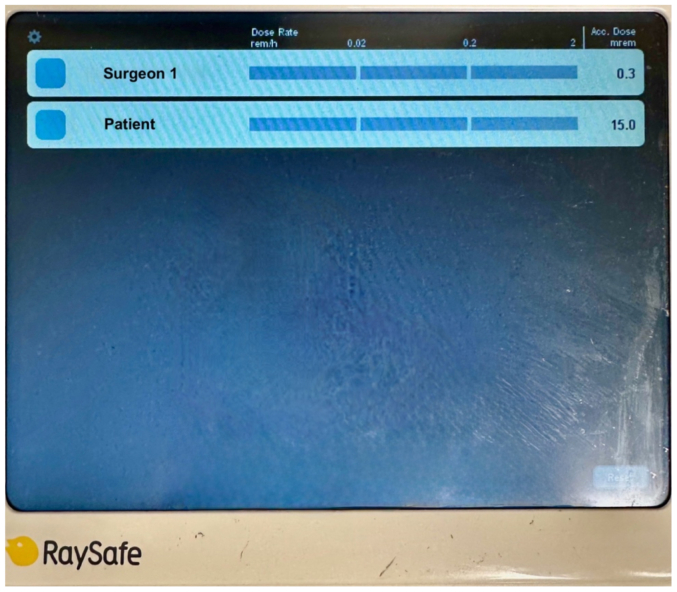
RaySafe real-time dosimeter demonstrating effective dose from scatter radiation during an endovascular aortic repair case.

Our patient is a 79-year-old woman who presented to our facility to undergo endovascular repair of a 5.4-cm infrarenal abdominal aortic aneurysm (Fig 1). Consent was obtained from the patient to use her case in this publication. She had a large inferior mesenteric artery and large flow lumen within the aneurysm so prophylactic embolization of the aortic sac was performed during this procedure. The Rampart Guardian device was used during this case to reduce radiation exposure to the surgical team. Real-time electronic dosimeters were used to measure the exposure of the primary operating surgeon and the patient during the procedure. The dosimeters were positioned on the left hip of the operator and near the left shoulder of the patient to measure scatter radiation (Fig 2). Case duration was 57 minutes with a total fluoroscopy time of 6.5 minutes. The estimated skin dose was 202 mGy. The operative surgeon received 0.3 mRem effective dose on a real time dosimeter (Fig 3). The patient received an effective dose of 15 mrem. The patient did well postoperatively and was discharged home the following day.

## Discussion

Radiation safety in the era of endovascular procedures is of utmost importance, particularly with increasing complexity and radiation exposure. Patient body habitus contributes to radiation exposure, and rising rates of obesity may increase radiation doses.^7^, ^8^, ^9^ Radiation exposure has untoward effects on the patient and surgical team, defined as stochastic and deterministic effects. Deterministic effects are dose dependent with direct cellular damage. Skin erythema akin to sunburn can be seen at 2000 mGy exposure and hair loss at 3000 mGy exposure.^10^ Stochastic effects are a more occult injury with damage to cellular DNA, which can lead to the development of malignancy.^11^ Although deterministic effects are dose dependent, the stochastic exposure-effect relationship is much more difficult to characterize. Regardless of demographic or case-specific factors, novel protection systems can decrease radiation dose and relieve constraints of traditional lead aprons and shields.

The Rampart System is a comprehensive radiation protection system designed to provide total body coverage for the team, enabling procedures with minimal reliance on lead aprons. Clinical evidence, including two randomized controlled trials, demonstrates ≤20-fold greater protection than traditional lead aprons and shields and approximately 99% reduction in scatter radiation.^4^^,^^5^

The Rampart Guardian is a full-body radiation shield system designed to minimize scatter for the team. The system is mobile at our facility and can be moved in conjunction with the patient during the procedure to make adjustments. The Rampart Bunker is an undertable radiation shield designed to reduce scatter exposure at the operator and assistant levels without restricting procedural access. The Rampart Shadow is a flexible radiation shield designed to be placed directly on the patient to block scatter radiation at its source. It is reusable and can be rolled for storage.

There are multiple stand or ceiling based radiation protection technologies available. The EggNest (Egg Medical, St Paul, MN) is a mounted system similar to Rampart, which has shown promising early results with radiation reduction for the entire team. Similar to Rampart, they have different configurations designed for different interventional suites. The Rampart Guardian has a trifold barrier that can be collapsed around the image intensifier to further decrease scatter to the anesthesia and operating team. Another strategy is to reduce the radiation dose directly using fusion imaging or with the use of intravascular ultrasound examination. However, with complex aortic repairs, radiation remains a necessity. Although our case was straightforward and with associated short operative and fluoroscopy times, we plan to evaluate the system during more complex procedures. Additionally, the patient was an ideal case with a body mass index of 18 and minimal difficulty with access. Future use of this system during less ideal situations will help to determine whether these results can be replicated. We plan to further evaluate exposure in the room with additional real-time radiation badges once these are obtained by our facilities.

Occupational radiation exposure remains a significant concern in interventional medicine, with chronic scatter exposure linked to cataract formation, thyroid disease, and potential malignancy despite adherence to ALARA principles. Traditional lead-based protective garments impose substantial ergonomic strain; Andrew et al^2^ reported that a 15-lb lead apron exerts approximately 300 lbs/sq in of pressure on vertebral discs. In the Society for Cardiovascular Angiography & Interventions 2023 Occupational Health Hazards Survey, 66% of operators reported musculoskeletal pain attributed to lead use, with 34% citing lumbar and 25% cervical spine injury, and nearly one in five respondents had taken a leave of absence owing to physical or mental occupational hazard or injury.^12^ These data underscore the need for alternative radiation protection systems that effectively reduce exposure while mitigating the physical burden of conventional shielding.

## Conclusions

Endovascular therapy in vascular surgery continues to grow with the concurrent advancement of medical devices to treat complex vascular pathology. Fluoroscopy is necessary to perform these procedures, but has both direct and indirect side effects to the patient and surgical team. Lead aprons and shields are often used to decrease exposure; however, this protection is often inadequate and places physical strain on the operator. Newer devices, such as the Rampart Guardian, have the ability to change the current endovascular paradigm and reduce radiation exposure in accordance with ALARA principles. Our experience has shown that real-time effective doses are extremely low using the Guardian system and may be the answer to decrease radiation exposure in complicated endovascular cases.

## Funding

None.

## Disclosures

None.
